# Supplementary material for: In Situ Recrystallization of Mesoporous Carbon–Silica Composite for the Synthesis of Hierarchically Porous Zeolites
Source: Materials (Basel). 2020 Apr 2;13(7):1640. doi: 10.3390/ma13071640 (PMC7178382; doi:10.3390/ma13071640)
Supplement: Supplementary file 1 [file materials-13-01640-s001.pdf]

Supplementary Materials: In Situ Recrystallization of Mesoporous Carbon–Silica Composite for the Synthesis of Hierarchically Porous Zeolites

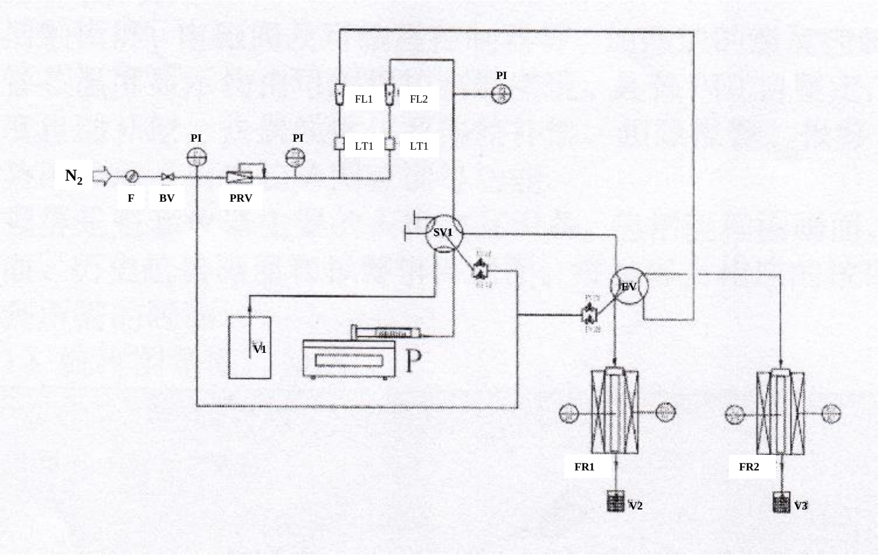

Figure S1. The schematic diagram of micro activity test unit. F: Filter valve; BV: Ball valve; PI: Pressure gauge; PVR: Pressure reducing valve; LT: Rotameter; FL: Flow control valve; SV: Filter valve; EV: Ball valve; P: Pressure reducing valve; V1: Rotameter; V2/V3: Flow control valve; FR: Reaction furnace.

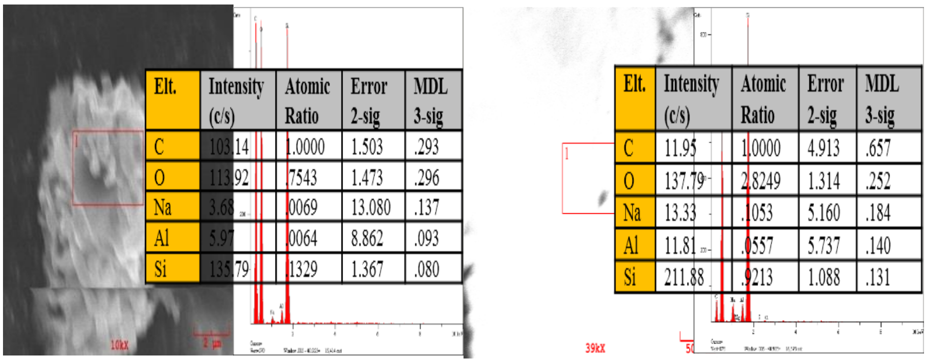

Elt.	Intensity (c/s)	Atomic Ratio	Error 2-sig	MDL 3-sig
C	103.14	1.0000	1.503	.293
O	113.92	7543	1.473	.296
Na	3.68	0069	13.080	.137
Al	5.97	0064	8.862	.093
Si	135.79	1329	1.367	.080

Elt.	Intensity (c/s)	Atomic Ratio	Error 2-sig	MDL 3-sig
C	11.95	1.0000	4.913	.657
O	137.79	2.8249	1.314	.252
Na	13.33	.1053	5.160	.184
Al	11.81	.0557	5.737	.140
Si	211.88	.9213	1.088	.131

Analysis Report: Image HPZ-CSi-8h

Figure S2. Elemental analysis with the EDS mounted on the SEM of HPZ-CSI-8h samples.

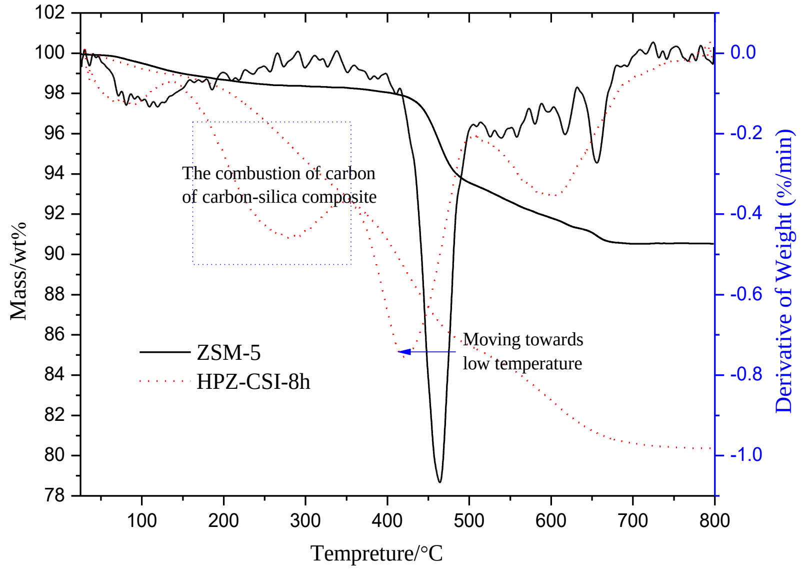

Figure S3. TG/DTA curves of HPZ-CSI-8h and ZSM-5 samples.

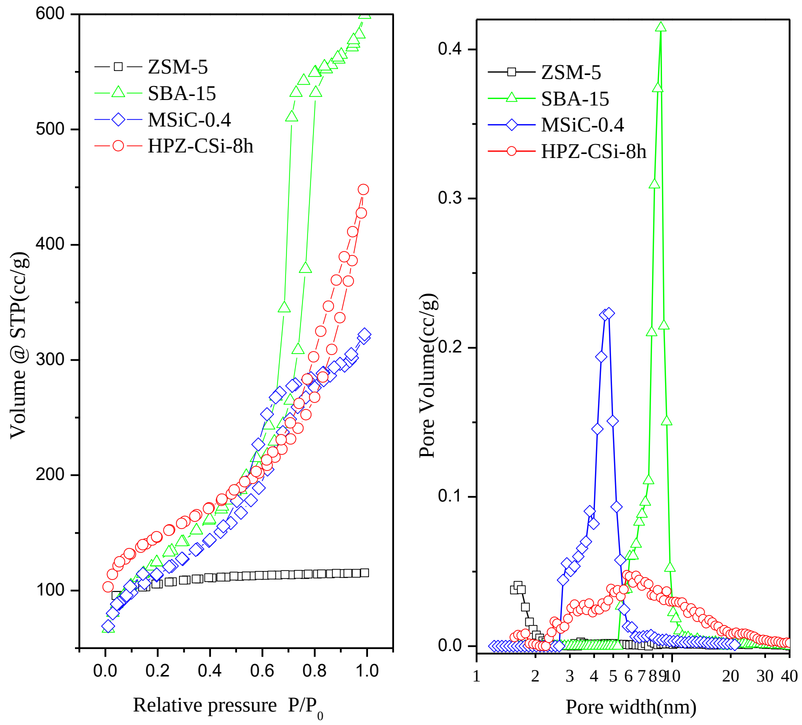

Figure S4. N_2 adsorption-desorption isotherms and pore size distribution of ZSM-5, SBA-15, MSiC-0.4 and HPZ-CSI-8h samples.

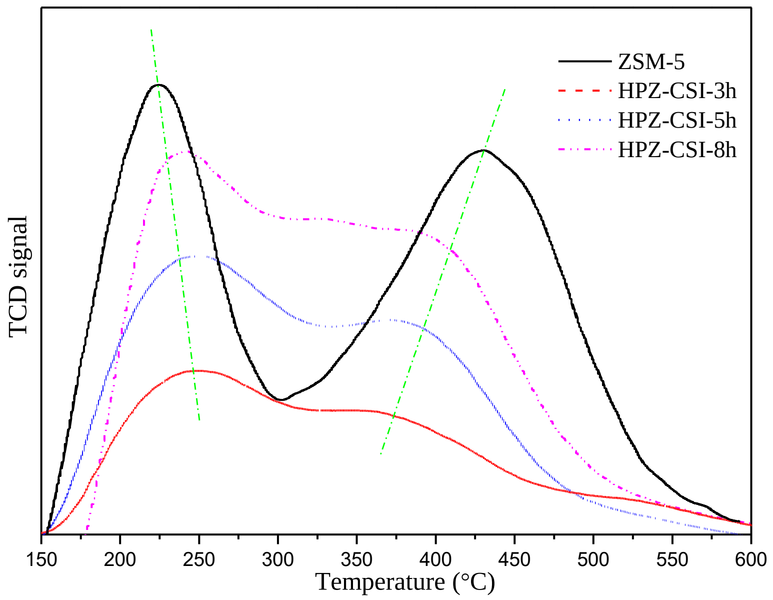

Figure S5. NH_3-TPD of HPZ-CSI-x and ZSM-5 samples.

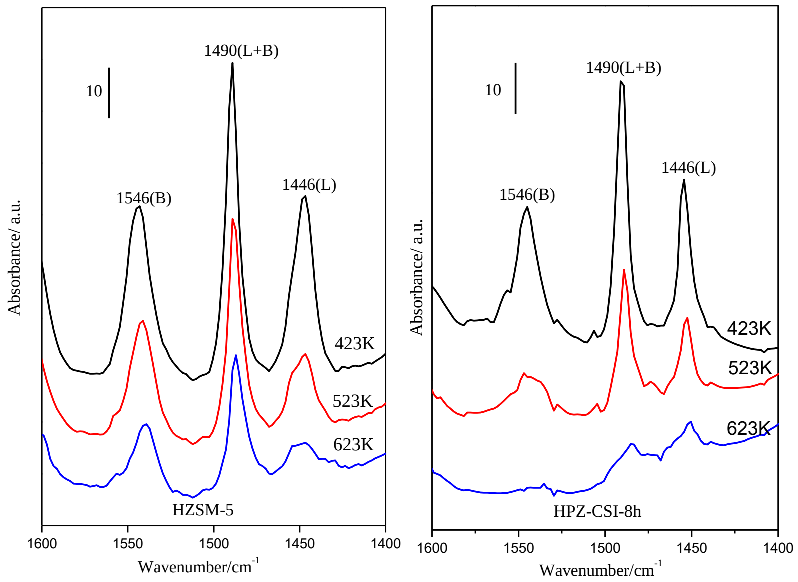

Figure S6. Pyridine-adsorbed IR spectra of HPZ-CSI-8h and ZSM-5 samples.
